# Changes in the bioelement content of summer and winter western honeybees (*Apis mellifera*) induced by *Nosema ceranae* infection

**DOI:** 10.1371/journal.pone.0200410

**Published:** 2018-07-25

**Authors:** Aneta A. Ptaszyńska, Marek Gancarz, Paul J. Hurd, Grzegorz Borsuk, Dariusz Wiącek, Agnieszka Nawrocka, Aneta Strachecka, Daniel Załuski, Jerzy Paleolog

**Affiliations:** 1 Department of Botany and Mycology, Institute of Biology and Biochemistry, Faculty of Biology and Biotechnology, Maria Curie-Skłodowska University, Lublin, Poland; 2 Institute of Agrophysics, Polish Academy of Sciences, Lublin, Poland; 3 School of Biological and Chemical Sciences, Queen Mary University of London, London, United Kingdom; 4 Laboratory of Environmental Biology and Apidologie, Institute of Biological Basis of Animal Production, Faculty of Biology, Animal Sciences and Bioeconomy, University of Life Sciences in Lublin, Lublin, Poland; 5 Department of Pharmacognosy, Ludwik Rydygier Collegium Medicum, Nicolaus Copernicus University, Bydgoszcz, Poland; 6 Department of Zoology, Ecology and Wildlife Management, Life Science University in Lublin, Lublin, Poland; University of North Carolina at Greensboro, UNITED STATES

## Abstract

Proper bioelement content is crucial for the health and wellness of all organisms, including honeybees. However, the situation is more complicated in these important pollinators due to the fact that they change their physiology during winter in order to survive the relatively harsh climatic conditions. Additionally, honeybees are susceptible to many diseases such as *nosemosis*, which during winter can depopulate an entire colony. Here we show that summer bees have a markedly higher content of important bioelements such as: Al, Cu, P, V, (physiologically essential); Ca, K, Mg, (electrolytic); Cr, Se, Zn, (enzymatic); As, Hg, (toxic). In contrast, a markedly higher content of: Fe (physiologically essential); Mn, Ni, (enzymatic); Cd (exclusively toxic) were present in winter bees. Importantly, *N*. *ceranae* infection resulted in an increased honeybee bioelement content of: S, Sr (physiologically essential) and Pb (exclusively toxic), whereas the *Nosema-*free worker-bees had higher amounts of B and Si (physiologically essential). We propose that the shortages of Fe, Mn, Ni, and Na observed in *Nosema-*infected bees, could be the reason for the higher mortality of *Nosema-*infected bees throughout overwintering. In addition, a shortage of bioelements such as B and Si may be a reason for accelerated aging in foragers that is observed following *N*. *ceranae* infection. Therefore, in winter, bioelement content was more strongly affected by *N*. *ceranae* infection than during summer. We found a strong correlation between the bioelement content of bees and seasons (summer or winter) and also with *Nosema* infection. We conclude that the balance of bioelements in the honeybee is altered by both seasonal affects and by *Nosema* infection.

## Introduction

*Nosema ceranae* infection (*nosemosis*) of western honeybees has been shown to cause numerous changes in the biology and physiology of the host. Due to pathogen-host relationships, the infected worker bees have increased hunger, lower foraging efficiency, altered behavior and impaired energy metabolism due to energetic stress [1–4]. Development of this obligate intracellular pathogen inside honeybee intestinal cells, also results in malnutrition most likely due to the microsporidian spore-made layer which completely covers the honeybee midgut [1,5,6]. This results in reduced food absorption, which combined with the effects mentioned above, may lead to higher forager bee mortality [1]. In contrast, *nosemosis* hardly affects intestinal microbiota and instead causes lesions in host glands and tissue. For instance, *nosemosis* can lead to partial damage of the rough endoplasmic reticulum, Golgi complex, mitochondria and pronounced myelin-like whorls of lysosome bodies in honeybee hypopharyngeal glands [5–7].

Current research on the bioelement composition of bee bodies have focused on using this information for ecological monitoring or as an assessment for the contamination of honeybee products [8–13]. Importantly, no studies have described changes in the host body bioelement composition due to *N*. *ceranae* infection or seasonal changes. We hypothesise that the physiological changes caused by the *Nosema* spp. in honeybees, may also seriously affect the distribution of bioelements in the host. It is symptomatic that an increase in the pollen content in a bee diet is on the one hand stimulated by the development of the *Nosema* spp., but on the other hand reduces its harmful effects on the host.

In the Biological System of the Elements described by Markertt et al. [14], and Skalny [15,16], bioelements are divided into four categories: physiologically essential, electrolytic, enzymatic and exclusively toxic elements. Physiologically essential elements (C, H, O, Si, P, S, N, B, F, Rb, Sr, Ba, Ti, Al, Br, Cs, Ge, and Te) are important constituents of the functional molecular structural elements of the cell, such as proteins, lipids, carbohydrates, and nucleic acids. Electrolytic elements (K, Na, Ca, Cl and Mg) are required for the maintenance of physiological potentials and defined osmotic conditions. Enzymatic elements (V, Cr, Mo, Mn, Fe, Co, Ni, Cu, Zn, Sn and Se), are mostly metal ions with catalytic functions in cell metabolism in the form of metal complexes. Exclusively toxic elements (Tl, Pb, Ga, Sb, In, Bi, Hg, and Cd) are lethal at low concentrations [14–16].

Currently, there are very few studies concerning the influence of bioelements during honeybee development [17], and more importantly, none describing changes in the host body bioelement composition due to microsporidian infection. Most bioelements are crucial for organismal development and wellbeing, therefore it is important to understand how *nosemosis* may alter the balance of bioelements in infected honeybees and contribute to honeybee health. Furthermore, since the honeybee host is a microenvironment for *N*. *ceranae* development, *N*. *ceranae* infection would be predicted to change the distribution of bioelements in the host. As well as the host bioelement needs, some bioelements would also be expected to be crucial for development of *N*. *ceranae* and therefore the overall balance of bioelements would be expected to be affected by *nosemosis*. In this present study, we show for the first time that *N*. *ceranae* infection changes the honeybee host’s bioelement composition and that these changes are seasonal.

## Material and methods

All protocols are available at 10.17504/protocols.io.qwedxbe

### Selection of the experimental honeybee colonies, sampling procedures and preliminary analysis of *Nosema* infection

The colonies originated from an apiary of 82 colonies at the Life Sciences University in Lublin, Poland (51°13'32.2"N 22°38'08.3"E). At the beginning of July 2015, 100 forager worker-bees (*Apis mellifera carnica*) from each colony were captured at the hive entrance. Forager bees were expected to have higher *Nosema* spp. infection level than younger bees [18] and were tested for *Nosema* infection (according to [19,20]). From the samples of 100-workers, 50 workers were removed from each and abdomens dissected, pooled, homogenized in distilled water and examined for the presence of *Nosema* spp. spores under an Olympus BX 61 light microscope. 10 of the remaining 50 workers were pooled, ground in distilled water and used for DNA analysis. If *Nosema* spp. spores were detected in a given sample, the colony was considered *Nosema-*infected (NI). In such cases, a heamocytometer was used to count the number of spores per worker bee. Colonies from which bees were found to contain no spores, were considered *Nosema-*free (NF). Consequently, five colonies which were *Nosema-*infected and five colonies which were *Nosema-*free were chosen as the experimental colonies and were subsequently kept in two locations 200 meters apart, separated by a hedge. This enabled the reduction of between-location drifting of forager-workers while at the same time, providing the colonies with access to the same food resources.

Two soil samples (in triplicate) were also taken in the immediate vicinity of the *Nosema-*free and *Nosema-*infected colonies. Bioelement content in the soil was expected to be related to the content in plants, and therefore also with the bee bioelement content [10,12].

In order to compare food resources, honey samples from *Nosema-*infected and *Nosema-*free colonies were analysed. Honey samples were taken during spring (May 2015), summer (August 2015) and winter (January 2016) and stored in sterile jars until further analysis.

In mid-July 2015, two pooled samples of approximately 100 worker bees, were collected in the evenings from the outer combs of each experimental colony (five *Nosema-*infected and five *Nosema-*free), to determine the bioelement composition in summer bees. Then, at the end of March 2016, after the bee winter cleansing flight, this procedure was repeated to determine the bioelement composition in overwintered bees. Fifty worker bees out of each pool were then subjected to DNA analysis and microscopy in order to confirm the *N*. *ceranae* infection status of each sample.

Both spring and summer samples of honey and also winter-food samples were taken from the colonies and stored for further bioelement analysis. The bioelement content of bee food is correlated with bioelement bee-body concentrations [8–10] and therefore this, along with the soil analyses [12], confirms that both the *Nosema-*infected and the *Nosema-*free bees used very similar bioelement resources. While over winter, all colonies were supplemented with the same high-quality food source of sugar (sucrose) syrup.

The *Nosema-*free and infected colonies were kept in two locations 200 meters apart and separated by a hedge. All apiary work were carried out in the same manner at both locations. Colonies had access to the same food resources and in autumn were supplemented sugar-water (2:1) syrup in preparation for overwintering.

All the procedures described above ensured that: (1) the colony *Nosema* infection status was determined by both microscopy and molecular analyses, (2) the sampled forager-workers had the closest contact with the colony external environment and suffered more from the *Nosema* infection than the younger nest bees, (3) bees were simultaneously tested for both *Nosema* status and for the bioelement content, (4) analysis of both soil and honey samples confirms that both the *Nosema-*infected and the *Nosema*-free bees used very similar nutrient and environmental resources with almost identical bioelement content.

#### DNA analysis

DNA was extracted from each of the pooled samples of homogenized worker bees as follows: 100 μl of each homogenate was added to 180 μl of lysis buffer and 20 μl of proteinase K and total DNA was isolated using the DNeasy Blood and Tissue Kit (Qiagen) according to the manufacturer’s instructions. Subsequently, each of the DNA samples was used as a template for detection of *N*. *apis* and *N*. *ceranae* 16S rDNA by PCR with *Nosema*-specific primers: 321-APIS for *N*. *apis* and 218-MITOC for *N*. *ceranae* [21].

#### Analysis of bioelement composition

Honeybee samples. The bioelement composition in each of the pooled worker bee samples was determined using Inductively Coupled Plasma Optical Emission Spectrometry (ICP-OES, iCAP Series 6500, Thermo Scientific, USA). Two sets of pooled bees (10 workers each) were mineralized in a Microwave Digestion System (Bergh of Speedwave, Eningen, Germany) by using optical, temperature and pressure monitoring of each sample during acid digestion in teflon vials (type DAP 100). The mineralized worker bee bodies were digested with 7 ml HNO_3_ (65% v/v) and 3 ml H_2_O_2_ (30% v/v). Each of the samples were performed in triplicate. Therefore 6 measurements were taken for each experimental colony, giving a total of 30 measurements for each bioelement (5 colonies x 2 pooled samples x 3 replicates).

Honey samples. In total, 12 honey samples were analysed: four spring honeys (two from NF and two from NI colonies), four summer honeys (two from NF and two from NI colonies) and four winter stores (two from NF and two from NI–colonies). The mineralization of each 0.5 g honey sample was conducted in a Microwave Digestion System (Bergh of Speedwave, Eningen, Germany) by using optical, temperature and pressure monitoring of each sample during acid digestion in teflon vials (type DAP 100). The mineralized honey samples were digested with 7 ml HNO_3_ (65% v/v) and 3 ml H_2_O_2_ (30% v/v). Each of the samples were performed in triplicate.

The mineralisation of honeybees and honey was as follows: 15 mins from room temperature to 140°C, 5 mins at 140°C, 15 mins from 140°C to 185°C, 10 mins at 185°C and then cooling down to room temperature. The pressure did not exceed 20 bars during mineralisation. After the mineralisation, the clear solution was cooled to room temperature and then transferred to 50 ml graduated flasks and filled with deionized water (ELGA Pure Lab Classic).

Soil samples. Six soil samples were also taken. Three were taken in the immediate vicinity of the colonies which were *Nosema-*free and similarly, the three from the *Nosema-*infected colonies. 0.5 grams of soil from each sample was digested in 8 ml of aqua regia, with 2 ml hydrofluoric acid in a high-pressure microwave digestion system (Berghof Speedwave, Eningen, Germany). Then the digested samples were made up to 50 ml with deionized water.

The operating conditions of the ICP-OES equipment were as follows: RF generator power of 1150 W, RF generator frequency of 27.12 MHz, coolant gas flow rate of 16 L·min^−1^, carrier gas flow rate of 0.65 L·min^−1^, auxiliary gas flow rate of 0.4 L·min^−1^, maximum integration time of 15 s, pump rate of 50 rpm, viewing configuration–axial, replicate– 3, flush time of 20 s.

The following multi-element stock solutions from Inorganic Ventures were used to prepare standards for all the analyses described above:

A) Analityk-46 for Cu, Fe, Mg, P, K, Na in 5% HNO_3_ (1000 μg/mL)

B) Analityk-47 for Al, As, Cd, Cr, Pb, Mn, Hg, Ni, Sc, Se, Sr, V, Zn in 10% HNO_3_ (100 μg/mL)

C) Analityk-83 for Ca, K, Mg, Na, P, S in 2% HNO_3_ (1000 mg/L)

D) Analityk prepared from single-element stock solutions for B, S, Si in 5% HNO_3_ (1000 mg/L)

E) CGMO1-1: Mo in H_2_O/tr. NH_4_OH (1000 μg/mL).

The bioelement symbols are compliant with the standards of the International Union of Pure and Applied Chemistry (IUPAC).

### Statistical analyses

Four pooled worker bee groups were analysed: summer *Nosema-*free (S-NF), summer *Nosema-*infected (S-NI), winter *Nosema-*free (W-NF) and winter *Nosema-*infected (W-NI). Tukey tests (one-way ANOVA, Statistica version 12.0, StatSoft Inc., USA) at the significance level of α = 0.05 were used to prepare the results presented in Table 1.

**Table 1:** The bioelement content [ng/mg] in *Nosema-*free and *Nosema*-infected honeybees collected in summer and winter.

	Summer *Nosema-*free bees	Summer *Nosema-*infected bees	Winter *Nosema-*free bees	Winter *Nosema-*infected bees		Statistical interpretation Symbol < or > means that a bioelement’s average is significantly lower or higher than the other bioelement average for p ≤ 0.05 Symbol = means that the difference between the averages of a given bioelement is not significant for p ≤ 0.05
Physiologically essential bioelements (mean ± SD; n = 30)						
Al	15.36 (SD = 1.244)	14.57 (SD = 1.216)	6.63 (SD = 0.191)		2.35 (SD = 0.145)	S-NF = S-NI; W-NF >W-NI; S-NF >W-NF; S-NI >W-NI
B	180.30 (SD = 13.828)	38.40 (SD = 2.723)	210.20 (SD = 17.243)		43.30 (SD = 2.847)	S-NF >S-NI; W-NF >W-NI; S-NF <W-NF; S-NI <W-NI
Cu	10.02 (SD = 0.813)	15.90 (SD = 0.129)	5.12 (SD = 0.114)		6.36 (SD = 0.128)	S-NF <S-NI; W-NF <W-NI; S-NF >W-NF; S-NI>W-NI
Fe	25.36 (SD = 1.147)	38.34 (SD = 2.205)	49.26 (SD = 2.130)		37.36 (SD = 2.931)	S-NF <S-NI; W-NF >W-NI; S-NF <W-NF; S-NI = W-NI
P	1865 (SD = 44.120)	2548 (SD = 53.020)	375.30 (SD = 6.345)		389.40 (SD = 13.660)	S-NF <S-NI; W-NF = W-NI; S-NF >W-NF; S-NI >W-NI
S	1146 (SD = 25.155)	2032 (SD = 31.120)	1668 (SD = 37,671)		1779 (SD = 40.600)	S-NF <S-NI; W-NF <W-NI; S-NF <W-NF; S-NI >W-NI
Si	1580 (SD = 49.000)	350.20 (SD = 21.656)	1309 (SD = 60.170)		110.60 (SD = 18.506)	S-NF >S-NI; W-NF >W-NI; S-NF >W-NF; S-NI >W-NI
Sr	4.35 (SD = 0.018)	7.26 (SD = 0.025)	2.26 (SD = 0.015)		8.39 (SD = 0.278)	S-NF <S-NI; W-NF<W-NI; S-NF >W-NF; S-NI <W-NI
V	0.1814 (SD = 0.041)	0.2321 (SD = 0.014)	0.0448 (SD = 0,017)		0.0336 (SD = 0.018)	S-NF <S-NI; W-NF = W-NI; S-NF **>**W-NF; S-NI >W-NI
Electrolytic bioelements (means ± SD; n = 30)						
Ca	421.40 (SD = 9.897)	497.20 (SD = 6.901)	398.00 (SD = 8.167)	420.70 (SD = 8.027)		S-NF <S-NI; W-NF <W-NI; S-NF >W-NF; S-NI >W-NI
K	3854 (SD = 80.040)	4411 (SD = 126.200)	2488 (SD = 50.831)	1868 (SD = 29.160)		S-NF <S-NI; W-NF >W-NI; S-NF >W-NF; S-NI >W-NI
Mg	356.00 (SD = 28.130)	470.70 (SD = 38.470)	246.60 (SD = 20.683)	207.10 (SD = 25.774)		S-NF <S-NI; W-NF >W-NI; S-NF>W-NF; S-NI >W-NI
Na	255.90 (SD = 28.13)	422.60 (SD = 14.960)	545.40 (SD = 30.231)	332.80 (SD = 25.441)		S-NF< S-NI; W-NF >W-NI; S-NF <W-NF; S-NI >W-NI
Enzymatic bioelements (means ± SD; n = 30)						
Cr	0.4961 (SD = 0.065)	0.5944 (SD = 0.027)	0.3738 (SD = 0.014)	0.2024 (SD = 0.019)		S-NF <S-NI; W-NF >W-NI; S-NF >W-NF; S-NI >W-NI
Mn	1.643 (SD = 0.176)	6.212 (SD = 0.237)	11.46 (SD = 0.125)	9.577 (SD = 0.173)		S-NF<S-NI; W-NF >W-NI; S-NF <W-NF S-NI <W-NI
Ni	0.2277 (SD = 0.026)	0.2927 (SD = 0.016)	2.106 (SD = 0.055)	0.4392 (SD = 0.065)		S-NF <S-NI; W-NF >W-NI; S-NF <W-NF; S-NI <W-NI
Se	1.096 (SD = 0.153)	1.556 (SD = 0.152)	0.6466 (SD = 0.037)	0.6677 (SD = 0.097)		S-NF <S-NI; W-NF = W-NI; S-NF >W-NF; S-NI >W-NI
Zn	55.54 (SD = 2.324)	71.40 (SD = 7.261)	34.53 (SD = 1.036)	37.36 (SD = 1.116)		S-NF <S-NI; W-NF <W-NI; S-NF >W-NF; S-NI> W-NI
Exclusively toxic bioelements (means ± SD; n = 30)						
As	0.3023 (SD = 0.336)	0.3868 (SD = 0.225)	0.0746 (SD = 0.052)	0.0560 (SD = 0.027)		S-NF = S-NI; W-NF = W-NI; S-NF >W-NF; S-NI >W-NI
Cd	0.0236 (SD = 0.004)	0.0435 (SD = 0.006)	0.0891 (SD = 0.007)	0.1881 (SD = 0.009)		S-NF <S-NI; W-NF >W-NI; S-NF <W-NF; S-NI <W-NI
Hg	0.1209 (SD = 0.029)	0.1547 (SD = 0.046)	0.0299 (SD = 0.011)	0.0224 (SD = 0.005)		S-NF <S-NI; W-NF = W-NI; S-NF >W-NF; S-NI >W-NI
Pb	0.4182 (0.058)	0.7096 (SD = 0.160)	0.4309 (SD = 0.049)	1.6200 (SD = 0.113)		S-NF< S-NI; W-NF <W-NI; S-NF <W-NF; S-NI <W-NI

Abbreviations: S-NF–summer *Nosema-*free honeybees. S-NI–summer *N*. *ceranae* infected honeybees. W-NF–winter *Nosema-*free honeybees. W-NI–winter *N*. *ceranae* infected honeybees.

Principal component analysis (PCA) was performed using the software package, Statistica (version 12.0, StatSoft Inc., USA), at the significance level of α = 0.05. The data were log-transformed, centred and standardised by bee group (i.e. S-NF, S-NI, W-NF, W-NI) but not by sample; thus, PCA was performed on the correlation matrix. The data matrix for PCA had four columns and 22 rows and the influences of two factors were considered: *Nosema* infection status (*Nosema-*infected or *Nosema-*free bees) and worker bee type (summer or winter). Consequently, the analysis was used to compare the multi-elemental stoichiometric relationships between bioelements and in this context, among worker bee groups: winter bees, summer bees, *Nosema-*free bees, and *N*. *ceranae* infected bees. The interactions between these factors were evaluated by two-way ANOVAs (Statistica version 12.0, StatSoft Inc., USA) performed separately for each bioelement. Correlations (interrelations) between respective bioelement content in bees were assayed on the correlations vectors at the PCA graph.

Differences in the bioelement content in the winter stores, spring and summer honeys were determined by comparing two one-way ANOVAs (p ≤ 0.05; honeys produced by *Nosema-*infected and *Nosema*-free bees) and two-way ANOVA (p ≤ 0.05 winter stores, summer, and spring honeys produced by *Nosema-*infected and *Nosema*-free bees) (Statistica version 12.0, StatSoft Inc., USA). These data are presented in S1 Table. Additionally, principal component analysis (PCA) was performed using the software package, Statistica (version 12.0, StatSoft Inc., USA), at the significance level of α = 0.05. The data were log-transformed, centred and standardised by honey or food group (i.e. SpH-NI spring honey made by *Nosema*-infected bees, SpH-NF spring honey made by *Nosema*-free bees, SH-NF summer honey made by *Nosema*-infected bees, SH-NI summer honey made by *Nosema*-free bees, WF-NF winter food stored by *Nosema*-infected bees, WF-NI winter food stored by *Nosema*-free bees) but not by sample; thus, PCA was performed on the correlation matrix. The data matrix for PCA had four columns and 22 rows and the influences of two factors were considered: *Nosema* infection status (*Nosema*-infected or *Nosema*-free bees) and spring or summer honey compared to winter stores. These data are presented in S1 and S2 Figs.

## Results

The infection status of the experimental colonies was confirmed by DNA analysis and revealed that all colonies considered *Nosema-*free had neither *N*. *apis* nor *N*. *ceranae* DNA, whereas *Nosema-*infected colonies were infected solely by *N*. *ceranae*. Colonies which had been found to be *Nosema-*free in summer 2015, were still *Nosema-*free in spring 2016, whereas the *Nosema-*infected colonies remained infected solely by *N*. *ceranae*. The infection level in the *Nosema-*infected colonies ranged from 4 x 10^6^ spores/bee in mid-July to 7 x 10^6^ spores/bee at the end of March. Changes in *Nosema* spp. infection level have also been previously reported by others [22–23] and are similar to changes observed for temperate regions of USA, Canada, and Germany [24–26].

*Nosema-*free and infected colonies had access to the same food supplies as evidenced by the very similar bioelement composition of honey taken from these experimental colonies (see Results: Bioelement content in honeys and winter stores, and Tables a-c in S1 Table). In addition, winter stores were made from feeding colonies in autumn with identical sugar:water (2:1) syrup. Therefore, honeybees that originated from either *Nosema-*free or infected colonies had access to the same food supplies and therefore the impact of *nosemosis* and seasonal changes on bioelement composition could be determined.

There were no differences between the bioelement content of the soil sampled near the *Nosema-*free or the *Nosema-*infected colonies, therefore data from these soil samples were averaged (Table d in S1 Table). In addition, there were no correlations between bioelement content in honeybees and in the apiary soil. Moreover, the experimental colonies were kept in relatively unpolluted environments because the soil content of toxic bioelements such as As, Cd, Hg, and Pb were low (Table d in S1 Table).

In the summer bees, markedly higher levels of the following bioelements were observed: Al, Cu, P, V (physiologically essential); Ca, K, Mg, (electrolytic); Cr, Se, Zn, (enzymatic); As, Hg, (exclusively toxic). In contrast, markedly higher levels of: Fe (physiologically essential); Mn, Ni, (enzymatic); and Cd (exclusively toxic) were observed in the winter bees. Therefore, the bee bioelement content differed markedly by season (summer vs winter bees) and independently of the *Nosema* infection status. Furthermore, summer bees consistently had much higher overall levels of bioelements. However, the *Nosema* infection status did influence the content of some bioelements independently of the bee type: *N*. *ceranae* infection increased bioelement contents of S, Sr (physiologically essential) and Pb (exclusively toxic), whereas the *Nosema-*free bees had higher amounts of B and Si (physiologically essential). Therefore, both experimental factors, i.e. bee type and *Nosema* infection status, influenced the content of most of the bioelements analyzed in our study (see Table 1, compare with S2 Table).

### Correlations between the bioelement content of worker honeybees

**Fig 1:**
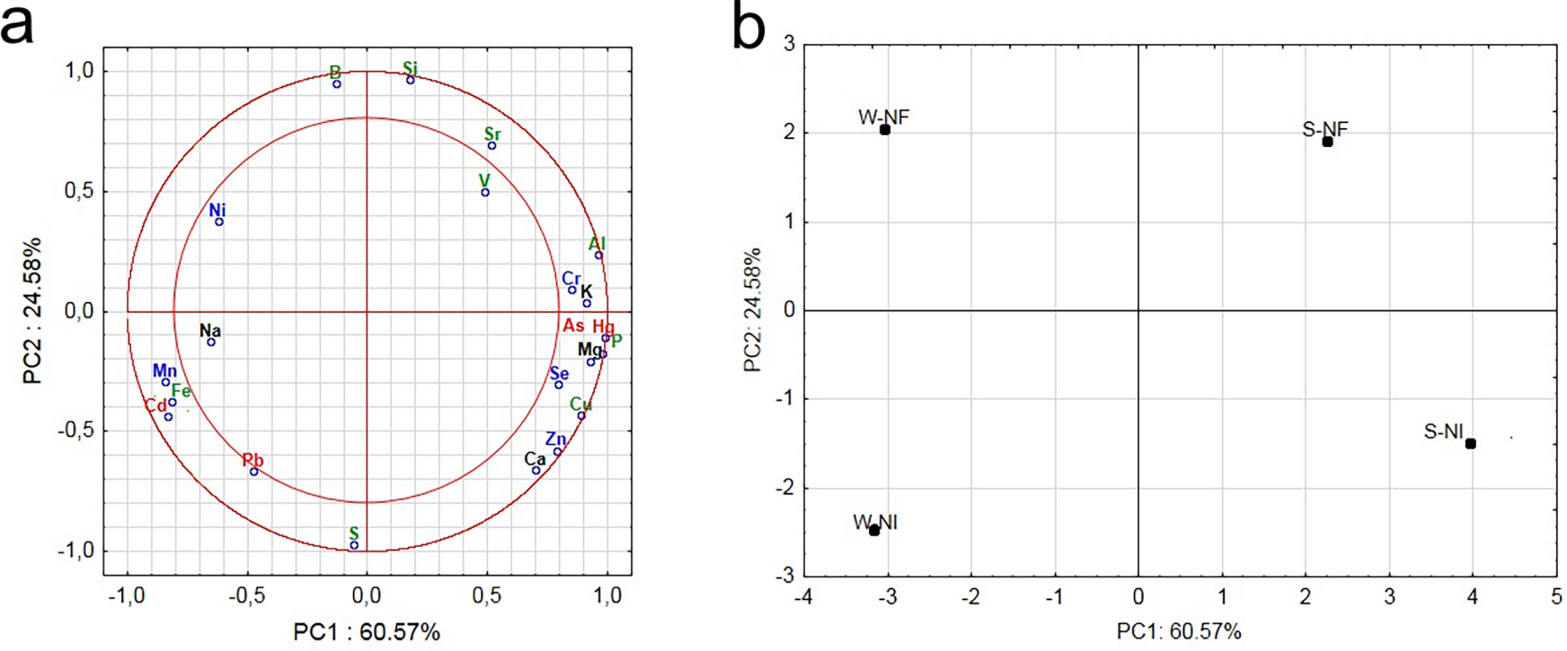
Principal component analysis (PCA) of twenty two bioelements for honeybees from different conditions. (a) A variable graph showing the position of the load vectors relative to the first two principal components; physiologically essential bioelements are marked in green (Al, B, Cu, Fe, P, S, Si, Sr, and V), electrolytic in black (K, Na, Ca, Cl, and Mg), enzymatic in blue (Cr, Mn, Se, Zn, and Ni) and exclusively toxic in red (Cd, Hg, Pb, and As). (b) The graph shows a strong correlation of bioelement content with seasons (summer, winter), and further with a *Nosema* infection.

For the physiologically essential elements, strong positive correlations (Fig 1A) were observed between concentrations of bioelement groups such as: B and Si, Sr and V. Simultaneously, the B and Si group was negatively correlated with the S. For the electrolytic elements, concentrations of Ca, K and Mg were positively correlated with each other (K with Mg strongly). A negative correlation was found between Na and the remaining electrolytic elements (K, Mg, Ca). For the enzymatic bioelements, positive correlations were observed among Cr, Zn, and Se, whereas there was a weak positive correlation between Mn and Ni. A strong negative correlation was found among enzymatic bioelements as elements belonging to these two groups; i.e. Cr, Zn, Se and Mn, Ni. For exclusively toxic elements, a strong positive correlation was found between As and Hg, as well as between Cd and Pb (Fig 1A).

In general, bioelements such as Ca, Zn, Cu, Se, Mg, P, K, Cr, Al, As, and Hg were the most closely correlated bioelement group. Negative correlations were observed between the bioelements belonging to this group and Na, Mn, Fe and Cd.

PCA resolved data into three major components which accounted for 100% of the variation and both figures i.e. Fig 1A and 1B should be consider simultaneously. Components PC1, PC2 and PC3 accounted for 60.57%, 24.58% and 14.85% of the variation, respectively. PC scores and loadings plots of PC1 versus PC2 are shown in Fig 1B. The significance of the bee type (i.e. summer or winter) was more pronounced and amounted to 61% of 22 bioelements (see PC1; Fig 1B), whereas *Nosema* infection status influenced only 25% of the bioelements (see PC2; Fig 1B). The greater significance associated with bee type rather than with *Nosema* infection status was also confirmed by ANOVA (two-way ANOVA, p ≤ 0.05; Supporting Information, S2 Table). Additionally, the ANOVA demonstrated that interactions between *Nosema* infection status and bee type contributed very significantly to the overall variance of bioelement content. PCA analysis (see Fig 1B) also revealed that interactions between bee type and *Nosema* infection status were important for 80% of the enzymatic bioelements (Cr, Mn, Se) and 75% of the electrolytic bioelements (Ni, Ca, K, Mg) but only for 30% of the physiologically essential bioelements (Fe, P, V) and 25% of the exclusively toxic bioelements (Hg). PCA also confirmed that differences between summer and winter bees were approximately two PC1 units larger in the *Nosema-*infected bees, than in the *Nosema-*free bees (Fig 1B). In contrast, differences between *Nosema-*infected and *Nosema-*free bees were approximately one PC2 unit larger in winter. Therefore, in winter, bioelement content was more strongly affected by *N*. *ceranae* infection than during summer.

### Bioelement content of honeys and winter stores

The bioelement content of honeys differ significantly by season. Compared to spring or summer honeys, winter stores had reduced levels of most bioelements presumably due to the fact that in autumn, colonies were supplemented with sugar:water syrup. With respect to spring honey, significant differences were detected in relation to winter stores for 14 out of the 22 bioelements (i.e. Al, As, Ca, Cr, Cu, Fe, Hg, K, Mg, Mn, Ni P, S, and Si, Table a in S1 Table). Finally, with respect to summer honey, differences were observed for 16 out of 22 bioelements (i.e. Al, As, Ca, Cd, Cr, Cu, Fe, K, Mg, Mn, Ni, P, S, Si, Sr, and Zn, Table b in S1 Table) in relation to winter stores.

When comparing spring honey and winter stores, strong positive correlations were observed among Cu, Ca, P, K, Mn, Mg, Fe, Al, S, Hg, Ag, Ni, Cr and a strong negative correlation between these bioelements and Pb and V (Figure a in S1 Fig). A comparison of summer honey and winter stores showed strong positive correlations among bioelements such as Mn, Zn, V, Cd, Al, and Na. Additional positive correlations between the Sr, S, Ca, K, P, Hg group and the Fe group and for the B, Ni, Si, and the Cr group were also observed. Strong negative correlations between Mn, Zn, V, Cd, Al, Na and Sr, S, Ca, K, P, Hg, Fe groups and also between B, Ni, Si, Cr and Cu were also observed (Figure a in S2 Fig).

PCA analyses revealed that the season played the major role in determining the bioelement content of both spring and summer honeys. For the spring honey vs winter stores, this component (PC1, Figure b in S1 Fig) enriched 75.16% (PC2, Figure b in S1 Fig). The impact of the *Nosema* status of the bees which made the spring honey was very weak. Generally, PCA analysis of the spring honey vs winter stores split data into three major components which accounted for the 100% of the variation (Figures a and b in S1 Fig, both figures should be considered simultaneously). PC1, PC2 and PC3 components accounted for 75.16%, 16.40% and 8.46% of the variation, respectively. The same correlations for the season and *Nosema* infection status on honey bioelement content were also confirmed by ANOVA (two-way ANOVA, p ≤ 0.05; Supporting Information, Table a in S1 Table).

Similar powerful effects of season were also observed for the comparison of summer honey and winter stores, where PC1 equalled 60.04%. In this case, the impact of the *Nosema* status of bees which made the summer honey was extremely weak. PCA analysis split data into three major components which accounted for 100% of the variation (Figures a and b in S2 Fig, both figures should be considered simultaneously). PC1, PC2, and PC3 components accounted for 60.04%, 27.83%, and 12.13% of the variation, respectively (Figure b in S2 Fig). The same correlations for the season and *Nosema* infection status on honey bioelement content was also confirmed by ANOVA (two-way ANOVA, p ≤ 0.05; Supporting Information, Table b in S1 Table).

## Discussion

### The seasonal impact on the bioelement content of honeybees and honeys

The seasonal impact (winter/summer bees) was more extensive and significant than the impact of the *Nosema* infection status. The content of most bioelements was higher in the summer bees. As summer worker bees have the possibility to forage, they can potentially complement the deficiency in bioelements, even when caused by *N*. *ceranae* infection.

Although we cannot exclude the possibility that higher metabolic rates of summer bees could contribute to higher bioelement concentrations, we still observe significantly higher levels of B, Fe, S, Na, Mn, and Ni in *Nosema-*free winter bees compared to *Nosema-*free summer ones. Therefore, it is highly likely that these bioelements play a crucial role in overwintering.

Overwintering bees have significantly reduced opportunities to forage, therefore during autumn they were supplemented a sugar:water syrup. Overwintering stress lead to decreased concentrations of the following bioelements: Ca, K, Mg, (electrolytic), Al, Cu, P, (physiologically essential); Cr, Se, Zn, (enzymatic), all of which have previously been linked to normal bee health [27–28]. Conversely, overwintering markedly increased levels of Fe (physiologically essential) and Mn, Ni, (enzymatic) in bees. Mn and Mg contents have previously been demonstrated to be the most consistent and therefore most likely to be regulated within narrowly defined limits in bees [9]. Furthermore, some bioelements have been demonstrated to be toxic to honeybees when present in excess, particularly Na [28]. Therefore, both the increase and decrease of particular bioelements in overwintered bees might be harmful. This is supported by recent research that demonstrates harmful, or even toxic effects, of a honeybee diet containing a low nutritional balance of bioelements [14,29].

Comparing data from PCA analyses for bees (Fig 1A, Fig 1B) and honey bioelement content (Supporting Information, Figures a and b in S1 and S2 Figs) showed that for both spring and summer honeys, the season played a major role. However in contrast, in both spring and summer the *Nosema* status of the bees that made the honey had negligible impact on the honey bioelement content, although, the summer honey bioelement content had a similar distribution pattern as the bee bioelement content (compare Fig 1A and Figure a in S2 Fig).

In summary, for both worker bees and the honeys they produce a strong correlation was observed between the bioelement content and season (summer or winter), with *Nosema* infection only very slightly influencing the bioelement content of the honeys.

### Impact of *N*. *ceranae* infection on the seasonal bioelement content of honeybees

Since our colonies of *Nosema-*infected and *Nosema-*free bees had access to the same food sources during summer and winter, we were able to investigate the impact of the *Nosema* infection.

Independent of the bee type (winter/summer), *N*. *ceranae* infection increased contents of S, Sr (physiologically essential), and Pb (exclusively toxic), whereas levels of B and Si (physiologically essential) were decreased. During summer, *Nosema-*infected bees have a deficit in certain bioelements (notably Al, B, and Si), which could lead to faster aging of a type observed in other organisms such as zebrafish, frogs and rats [30]. Boron deprivation disrupts embryonic development and caused abnormal development of the gut [31]. An absence of either Si or B has been shown in other organisms to increase susceptibility to pathogens [30–31]. Therefore, the *N*. *ceranae* development connected with shortages of B and Si could make bees more susceptible to other infection and may result in honeybee colony depopulation commonly observed after *N*. *ceranae* infection.

On the other hand, *N*. *ceranae* infection during summer did not drastically change the concentration of bioelements important for proper apian metabolism. During summer, bees are able to forage and gain access to a variety of food resources, which may help them compensate for deficiencies caused by *Nosema* infection. Previous research has revealed that the level of *Nosema* infection is higher among bees supplemented with good quality pollen than in bees supplemented with poor quality pollen but at the same time, longevity of worker bees is increased in bees which are supplemented with a well-balanced diet [13,28, 32–33]. Consequently, bees can tolerate higher *Nosema* infection levels, presumably due to better nutrition.

During overwintering, *Nosema-*free bees had increased levels of bioelements that are linked to fitness (Al, B, Fe, Si, K, Mg, Na, Cr, Mn and Ni) compared to *Nosema-*infected winter bees (Table 1). High levels of K and Mg are required by other insects, and therefore also probably by honeybees, for proper development [4,14,20,27–28]. Mg is a co-factor for many physiological processes [27,34] and low concentrations of K are found to be very harmful for bees, therefore K and Mg are commonly used as supplements for bee nutrition [28,34,35]. High concentrations of Fe and Mg are more important for older foraging bees; in particular, Fe is necessary for orientation of the forager bees in relation to the earth’s magnetic field [28]. Studies by Charbonneau et al. [36] indicated that *Nosema* infection does not have any effects on bee learning or memory, therefore disturbance in Fe balance could cause bee losses due to an inability to return to the hive.

Clearly *nosemosis* disturbs the balance of many bioelements in honeybees. A shortage of numerous essential bioelements could clearly be a cause of the higher mortality seen in *Nosema-*infected bees overwinter. In summer, the differences between the *Nosema-*infected and *Nosema-*free worker-bees is not as pronounced as that seen in winter (i.e. *N*. *ceranae* infection perturbs the balance of bioelement metabolism more severely in winter) and the more significant differences in the bioelement content between summer and winter bees solely within the *Nosema-*infected bees confirms this hypothesis.

### Influence of *N*. *ceranae* development on the bioelement content associated with key biochemical pathways

During *N*. *ceranae* development, energy is consumed directly from the ATP produced by the honeybee host via newly formed spores. Therefore, an infection could clearly disturb all physiological processes in the host. Infected worker bees may compensate for a loss of bioelements by accumulating more of them from the external environment while foraging, in order to cope with the increased demand associated with the infection. Conversely, during winter, honeybees cannot easily compensate for shortages in bioelement content by foraging. In our study, winter bioelement content was measured in colonies which were *N*. *ceranae* infected for at least one year, therefore honeybees from these colonies still had the potential to gather more bioelements during foraging over the summer. The observed excess of bioelements might have been induced by over-compensation due to losses caused by *N*. *ceranae* infection. We would have therefore predicted a more serious imbalance if the infection had taken place in autumn when it would have been too late to effectively replace bioelements via foraging. In comparison to *Nosema-*free bees, higher quantities of physiologically essential bioelements such as: Cu, P, S, and Sr were observed in *Nosema-*infected worker bees during summer and winter. P and S are important components of nucleic acids, proteins, phospholipids of cell membranes and therefore play a crucial role in energy generation and growth, which may explain their increase in infected bees.

Furthermore, during summer, higher content of all the electrolytic bioelements (Ca, K, Mg, and Na) was observed in the *Nosema-*infected bees in comparison to *Nosema-*free bees. Conversely, in winter bees, the *Nosema-*free bees had more electrolytic elements than the *Nosema-*infected ones (with the exception of Ca). K, Mg, and Na are the bioelements connected to regulating osmotic pressures and acid-base equilibriums. These bioelements also play important roles in water metabolism and energy biotransformation [37]. Therefore, post-infection shortages could drastically reduce bee survival during overwintering.

During summer, the content of enzymatic bioelements such as: Cr, Mn, Ni, Se, and Zn increased after an *N*. *ceranae* infection. In contrast, a decrease in Cr, Mn, and Ni contents were observed in the *Nosema-*infected bees during winter. Mn is a cofactor for many enzymes and is also involved in the metabolism of sugars, fats and proteins. It is also necessary for proper brain and muscle function and contributes to the hardness and abrasive resistance qualities of chitin [38]. After an infection, honeybees have to forage more actively due to hunger induced by *N*. *ceranae* development, therefore, muscles and brain must remain more active in comparison with uninfected bees and thus Mn could be accumulated by bees in response to the *N*. *ceranae* infection. Conversely, Mn shortages are observed after an *N*. *ceranae* infection during winter. This reduction may be explained by the fact that the *Nosema* spp. endospores contain chitin and protein [39], in which Mn metabolism is necessary. Furthermore, a shortage in Mn can cause growth disorders and diminish reproductive functions in vertebrates and crustaceans [40], which could explain the reduction in egg lying observed in *Nosema-*infected queens and consequently, supersedure after the *Nosema* spp. infection [41]. How *Nosema* infection impacts queen bees is still under investigation, but the main impact of infection may manifest more significantly in workers. The most significant *Nosema*-induced changes were observed in the winter bees, in particular the content of Ni, which was reduced 4.8-fold. Ni is a cofactor for numerous enzymes such as: glyoxalase I, acireductone dioxygenase, superoxide dismutase, [NiFe]-hydrogenase, acetyl-coenzyme A synthase/decarbonylase, methyl-coenzyme M reductase, carbon monoxide dehydrogenase and lactate racemase [42]. During winter, as with many other resources, Mn and Ni are involved in *N*. *ceranae* development, as well as in the response of the worker bee to the *Nosema*-caused pathological processes. Again, overwintering bees are not able to compensate for these shortages from the external environment via foraging. Similarly, Cr, which plays a role in glucose metabolism [43–44], is reduced in *Nosema-*infected worker bees. When *N*. *ceranae* multiply in honeybee intestines, all supplies of this bioelement can be consumed by the pathogens [45]. The highest amounts of Ni and Mn were found in winter *Nosema*-free worker bees and could be connected with ensuring enzymes that were protected from denaturation while overwintering. Since *N*. *ceranae* infection would be expected to drastically disturb this process, and *Nosema-*infected bees contain smaller amounts of Ni and Mn, this may explain the higher mortality of infected bees during winter.

The *N*. *ceranae* infection also caused significant changes in physiologically essential bioelements, such as B and Si, which were much lower in *Nosema-*infected bees (Table 1). B is required more in active summer worker bees as it interacts with molecules such as riboflavin, vitamin B6, coenzyme A, vitamin B-12, and nicotinamide adenine dinucleotide (NAD^+^) [46–48]. Si compounds, as well as B, can stimulate the growth of a large range of fungi [31,49], which may include those from the *Nosema* genus. Consequently, *N*. *ceranae* obtains these bioelements directly from apian tissues, which would be difficult to compensate for in overwintering bees. Furthermore, B and Si are also involved in animal aging and the demand for these bioelements is greatest in very young organisms [50]. Thus, one could postulate that *N*. *ceranae* infection could cause premature aging of worker-bees and shorten life expectancy [51].

### Influence of *N*. *ceranae* infection on the bioelement content associated with the anti-inflammatory response

Physiologically essential bioelements such as Sr and Cu, the enzymatic bioelement Zn, and the electrolytic bioelement Ca, are connected with the anti-inflammatory response [52–53] and *nosemosis* could potentially induce inflammation in honeybees. Moreover, Cu has antimicrobial activity against a wide range of microorganisms including fungi [54]. Furthermore, Zn has a possible protective effect on lipid peroxidation [55–56]. Therefore, honeybees may increase the concentration of Ca, Sr, Zn and Cu in order to protect against the harmful effects of *N*. *ceranae* infection, independently of the bee type. Recovery cases of mildly infected honeybee colonies have been reported [7,57]. Consequently, one can propose that increasing the concentration of these bioelements after *N*. *ceranae* infection may play a role in such recovery.

In summer bees, Fe content was greater in the *Nosema-*infected than in the *Nosema-*free worker bees, whereas in the winter bees, *Nosema-*infected worker bees had less Fe than the *Nosema-*free ones. Hence, the *N*. *ceranae* infection clearly influenced the Fe content in the context of the bee type (winter/summer). Not only is Fe necessary for bees’ orientation, but it is also accumulated in granules in the trophocytes of fat bodies in maturing bees, and is present in the hemolymph. Fe availability and its presence in the hemolymph is regulated by ferritin, a protein whose main function is iron transport, but it also plays a role in the insect immune response and the protection against oxidative challenges [28,37,58–59]. During the summer months, the energy resources of foragers are mainly expended on pollen and nectar collecting, with the result that individual timmunity is reduced [60]. Furthermore, foragers are more exposed to oxidative stress due to flying [61]. In response to these seasonally altered physiological processes interacting with the *N*. *ceranae* infection, forager honeybees could increase the accumulation of Fe from the external hive environment, particularly in late summer [14]. In contrast, overwintering *Nosema-*infected bees cannot forage and hence cannot compensate for a shortage of Fe in the same way.

In summer bees, Al content was similar in both the *Nosema-*infected and *Nosema-*free bees. However, in the winter bees, Al content was almost one third lower in the *Nosema-*infected bees compared to the *Nosema-*free ones (Table 1). A similar mechanism to the one suggested for Fe accumulation might be responsible because Al has the ability to increase antioxidant enzyme activity, which may be crucial for bees during *N*. *ceranae* winter development [62].

### Exclusively toxic bioelements

Exclusively toxic bioelements are lethal at even low concentrations [15–17]. Overabundance of Cd interferes with Ca and Zn metabolism and can damage the nervous system as can Hg, Pb, and As [63]. Moreover, Pb and Hg can degrade proteins, reduce enzyme activity and cause damage to cell membranes [64–65]. Furthermore, Pb damages reproductive organs [66] and its accumulation could be one of the causes for supersedure of the queen following *N*. *ceranae* infection [67–68]. Pb also affects the metabolism of crucial bioelements such as Fe, Ca, Cu, Mg, and Zn [63,69], which would in turn disturb the biological metabolism of the cell. In summer, *Nosema-*infected worker bees have higher concentrations of Cd, Hg, Pb, and As than *Nosema-*free bees. This could be related to their increased foraging activity due to *N*. *ceranae* infection. This increased accumulation of toxic bioelements in the bodies of the *Nosema-*infected bees would clearly cause harmful side effects.

Comparing the contents of Cu, Zn, Pb, Cd, Ni, Mn and Fe in honeybee bodies in our study to that reported by Zhelyazkova [70], confirmed that bees in our research originated from a region almost free of industrial pollution. This is in agreement with the low bioelement content of the soil collected from the apiary (Table d in S1 Table). This is important since the toxic bioelement content of soil manifest in the bioelement content of plants, and consequently in bee food, bodies and products [8,10,12]. Therefore importantly, the results of our studies are not due to toxic bioelement contamination from the environment. In both *Nosema-*infected and *Nosema-*free bees, As and Hg levels declined, whereas Cd and Pb concentrations increased as a result of overwintering. This decrease in As and Hg levels might be explained by low exposure of bees to the influences of the external environment during overwintering or due to unknown physiological mechanisms which prevent accumulation of these bioelements to toxic levels. The differences in behavioural mechanisms (i.e. water, nectar, and pollen foraging) might explain the differences observed for *Nosema-*infected and non-infected. colonies. Therefore, it would be very interesting to repeat this study in a more polluted environment or by using artificially contaminated food with highly *Nosema-*infected colonies, in order to study these phenomena.

## General remarks, conclusions and further research perspectives

Our studies have shown for the first time that *N*. *ceranae* infection results in profound changes in the bioelement content of the host honeybee, and that these changes often are different in winter and summer. Interaction between the N. *ceranae* infection and overwintering stress was particularly harmful for honeybee bioelement sequestration. These complex phenomena require further research in order to be fully delineated because disorders in bioelement metabolism are likely to be very important to honeybee health and survival during *Nosema* infection. The harmful side effects of *N*. *ceranae* infection on bioelement content and subsequently honeybee physiology might be an important cause of colony depopulation.

Combating *Nosema* spp. infection requires the activation of many host physiological mechanisms and would therefore be expected to require an increase in many important bioelements. In summer, foragers are able to compensate by increasing bioelement concentrations from the environment. In contrast, overwintering bees have little or no access to external nutrient resources, therefore *Nosema* spp. infection would cause a significant reduction in host bioelement concentration. Given this, the routine supplementation of bioelements to diets fed to winter *N*. *ceranae* infected honeybees might be considered a useful potential treatment. The content of most bioelements is strongly connected to each other (Fig 1A, especially the group of: Al, Ca, Cu, Cr, K, Mg, Se, V, Zn), therefore the alteration in levels of one could change the balance of all the others, and hence influence other important physiological processes that are not directly associated with the limiting the bioelement. The results presented here suggest that infection of honeybees by *N*. *ceranae* is complex and fundamentally affects host physiology in ways that were not previously apparent.

Given the importance of a balanced bioelement metabolism to honeybee health as well as the necessity for bees to obtain bioelement-rich nutrients from their foraging resources, modern monocultures could also hamper bioelement metabolism. Monoculture agriculture drastically diminishes pollen diversity [33–35], which may potentially lead to a destabilization of bioelement sequestration. Single-species crop plantations do not allow for the gathering of nutrients in adequate proportions and this could lead to a stoichiometric mismatch of bioelements [29]. Our current study has shown that *N*. *ceranae* infection may compound this phenomenon, and consequently may cause faster and more significant honeybee colony depopulation.

Our novel findings concerning the importance of apian bioelements improves our understanding of the factors that determine their balance and shortfalls or excesses. These findings may also have practical implications such as routine dietary supplementation and in addition, could help stimulate the development of apiculture methods that alleviate bioelement nutrient shortfalls in honeybees.

## Supporting information

S1 Table The bioelement content [ng/mg] in honey and soil samples. Statistica (version 12.0, StatSoft Inc., USA), at the significance level of α = 0.05. NI–*N*. *ceranae*-infected honeybees. NF–*N*. *ceranae*-free honeybees. Typed in bold–data differ significantly. **Table a in S1 Table. The results of comparison of bioelement content for the spring honey, the winter stores and *Nosema* infection status of bees which made the honey**. **Table b in S1 Table. The results of comparison of bioelement content for the summer honey, the winter stores and *Nosema* infection status of bees which made the honey. Table c in S1 Table. The bioelement content in the winter stores. Table d in S1 Table. The bioelement content in the apiary soil.** Samples taken near NI and NF colonies have not differ (were almost identical). Therefore. only the total averages were shown in this case.
(DOCX)

S2 Table Results of the two-way ANOVA, factors: *Nosema* infection status and worker bee type. Statistica (version 12.0, StatSoft Inc., USA), at the significance level of α = 0.05. Factors: *Nosema* infection status and bee type. NHS–*Nosema* health status (*Nosema-*infected *vs*. *Nosema-*free). WBT–worker bee type (summer *vs*. winter). WBT*NHS–interaction of the *Nosema* infection status vs worker-bee type. Insignificant effects–printed in bold type.
(DOCX)

S1 Fig Principal component analysis (PCA) of twenty two bioelements for the spring honey from different conditions. SpH-NI spring honey made by *Nosema-*infected bees, SpH-NF spring honey made by *Nosema-*free bees, WF-NF winter food stored by *Nosema-*infected bees, WF-NI winter food stored by *Nosema-*free bees. (a) A variable graph showing the position of the load vectors relative to the first two principal components; physiologically essential bioelements are marked in green (Al, B, Cu, Fe, P, S, Si, Sr, and V), electrolytic in black (K, Na, Ca, Cl, and Mg), enzymatic in blue (Cr, Mn, Se, Zn, and Ni) and exclusively toxic in red (Cd, Hg, Pb, and As). (b) The graph shows a strong correlation of bioelement content with seasons (summer, winter), and further with a *Nosema* infection.
(TIF)

S2 Fig Principal component analysis (PCA) of twenty two bioelements for the summer honey from different conditions. SH-NF summer honey made by *Nosema-*infected bees, SH-NI summer honey made by *Nosema-*free bees, WF-NF winter food stored by *Nosema-*infected bees, WF-NI winter food stored by *Nosema-*free bees. (a) A variable graph showing the position of the load vectors relative to the first two principal components; physiologically essential bioelements are marked in green (Al, B, Cu, Fe, P, S, Si, Sr, and V), electrolytic in black (K, Na, Ca, Cl, and Mg), enzymatic in blue (Cr, Mn, Se, Zn, and Ni) and exclusively toxic in red (Cd, Hg, Pb, and As). (b) The graph shows a strong correlation of bioelement content with seasons (summer, winter), and further with a *Nosema* infection.
(TIF)
