# Supplementary material for: Changes in the bioelement content of summer and winter western honeybees (Apis mellifera) induced by Nosema ceranae infection
Source: PLoS One. 2018 Jul 25;13(7):e0200410. doi: 10.1371/journal.pone.0200410 (PMC6060561; doi:10.1371/journal.pone.0200410)
Supplement: S1 Table — Statistica (version 12.0, StatSoft Inc., USA), at the significance level of α = 0.05. NI–N. ceranae-infected honeybees. NF–N. ceranae-free honeybees. Typed in bold–data differ significantly. Table a in S1 Table. The results of comparison of bioelement content for the spring honey, the winter stores and Nosema infection status of bees which made the honey. Table b in S1 Table. The results of comparison of bioelement content for the summer honey, the winter stores and Nosema infection status of bees which made the honey. Table c in S1 Table. The bioelement content in the winter stores. Table d in S1 Table. The bioelement content in the apiary soil. Samples taken near NI and NF colonies have not differ (were almost identical). Therefore. only the total averages were shown in this case. (DOCX) [file pone.0200410.s001.docx]

**S1 Table. The bioelement content [ng/mg] in honey and soil samples.**

**S1a Table. The results of comparison of bioelement content for the spring honey, the winter stores and *Nosema* infection status of bees which made the honey**.

|  | Spring honey | | | | One-way ANOVA results for NI vs NF | | Winter food | | | | Two-way ANOVA results for the comparison of an influence of a season and a bee *Nosema* status on honey bioelement contents | | | |
| --- | --- | --- | --- | --- | --- | --- | --- | --- | --- | --- | --- | --- | --- | --- |
|  | NI | | NF | |  |  | NI | | NF | | Two-way ANOVA results for season (spring honey vs winter stores) | | Two-way ANOVA results for *Nosema* status (spring honey produced by *Nosema-infected* vs *Nosema*-free bees) | |
|  | Mean | SD | Mean | SD | F | p | Mean | SD | Mean | SD | F | p | F | p |
| Al | 5.209 | 0.050 | 5.539 | 0.020 | 1.171 | 0.340 | 3.764 | 0.449 | 3.931 | 0.551 | 78.260 | **0.000** | 2.072 | 0.174 |
| As | 0.207 | 0.044 | 0.217 | 0.093 | 0.705 | 0.448 | 0.147 | 0.054 | 0.134 | 0.024 | 8.204 | **0.013** | 0.005 | 0.943 |
| B | 20.337 | 0.206 | 27.523 | 0.310 | 1118.195 | **0.000** | 24.350 | 0.527 | 23.935 | 0.179 | 0.040 | 0.845 | 10.105 | **0.007** |
| Ca | 145.600 | 0.173 | 144.767 | 1.007 | 2.388 | 0.197 | 83.715 | 1.302 | 83.350 | 0.608 | 23243.623 | **0.000** | 2.195 | 0.162 |
| Cd | 0.034 | 0.006 | 0.040 | 0.007 | 0.046 | 0.841 | 0.044 | 0.007 | 0.036 | 0.005 | 0.796 | 0.388 | 0.012 | 0.914 |
| Cr | 0.763 | 0.106 | 0.834 | 0.007 | 2.433 | 0.194 | 0.663 | 0.018 | 0.657 | 0.007 | 33.947 | **0.000** | 1.860 | 0.196 |
| Cu | 0.211 | 0.020 | 0.196 | 0.011 | 0.638 | 0.469 | 0.164 | 0.017 | 0.182 | 0.021 | 10.785 | **0.006** | 0.022 | 0.885 |
| Fe | 7.398 | 0.064 | 7.815 | 0.374 | 1.850 | 0.245 | 4.332 | 0.060 | 4.234 | 0.081 | 983.548 | **0.000** | 2.268 | 0.156 |
| Hg | 0.416 | 0.055 | 0.424 | 0.057 | 0.001 | 0.972 | 0.346 | 0.063 | 0.366 | 0.026 | 8.078 | **0.014** | 0.420 | 0.528 |
| K | 1007.733 | 18.655 | 998.967 | 4.366 | 0.607 | 0.480 | 182.950 | 8.719 | 182.500 | 7.991 | 28634.133 | **0.000** | 0.903 | 0.359 |
| Mg | 18.333 | 0.055 | 18.963 | 0.225 | 2.112 | 0.220 | 12.075 | 0.254 | 12.350 | 0.067 | 4896.662 | **0.000** | 24.206 | **0.000** |
| Mn | 0.603 | 0.011 | 0.617 | 0.004 | 0.965 | 0.382 | 0.229 | 0.006 | 0.224 | 0.008 | 8122.176 | **0.000** | 1.037 | 0.327 |
| Na | 3.213 | 2.628 | 5.267 | 1.059 | 1.577 | 0.278 | 5.003 | 0.540 | 5.666 | 0.275 | 3.262 | 0.094 | 5.027 | **0.043** |
| Ni | 0.565 | 0.052 | 0.601 | 0.066 | 0.086 | 0.783 | 0.462 | 0.009 | 0.466 | 0.003 | 47.085 | **0.000** | 1.311 | 0.273 |
| P | 70.937 | 0.733 | 69.883 | 0.015 | 3.591 | 0.131 | 28.303 | 0.335 | 28.183 | 0.203 | 38429.473 | **0.000** | 7.439 | **0.017** |
| Pb | 0.548 | 0.176 | 0.534 | 0.252 | 0.237 | 0.652 | 0.620 | 0.183 | 0.611 | 0.200 | 0.704 | 0.417 | 0.017 | 0.899 |
| S | 44.137 | 0.730 | 44.237 | 0.206 | 1.890 | 0.241 | 27.815 | 2.086 | 29.613 | 2.486 | 349.476 | **0.000** | 1.314 | 0.272 |
| Se | 0.348 | 0.053 | 0.406 | 0.056 | 0.006 | 0.943 | 0.316 | 0.080 | 0.360 | 0.101 | 1.281 | 0.278 | 2.209 | 0.161 |
| Si | 107.500 | 0.721 | 133.767 | 0.153 | 3809.472 | **0.000** | 102.850 | 1.103 | 101.175 | 1.352 | 22.795 | **0.000** | 9.939 | **0.008** |
| Sr | 0.791 | 0.012 | 0.775 | 0.006 | 0.655 | 0.464 | 0.808 | 0.028 | 0.765 | 0.009 | 0.184 | 0.675 | 12.600 | **0.004** |
| V | 0.411 | 0.135 | 0.468 | 0.158 | 0.048 | 0.837 | 0.528 | 0.078 | 0.553 | 0.109 | 3.746 | 0.075 | 0.622 | 0.445 |
| Zn | 3.090 | 0.035 | 3.985 | 0.008 | 1.117 | 0.350 | 4.485 | 0.809 | 4.036 | 1.192 | 3.382 | 0.089 | 0.322 | 0.580 |

**S1b Table. The results of comparison of bioelement content for the summer honey, the winter stores and *Nosema* infection status of bees which made the honey.**

|  | Summer honey | | | | One-way ANOVA results for NI vs NF | | Winter food | | | | Two-way ANOVA results for the comparison of an influence of a season and a bee *Nosema* status on honey bioelement contents | | | |
| --- | --- | --- | --- | --- | --- | --- | --- | --- | --- | --- | --- | --- | --- | --- |
|  | NI | | NF | |  |  | NI | | NF | | Two-way ANOVA results for season (summer honey vs winter stores) | | Two-way ANOVA results for *Nosema* status (summer honey produced by *Nosema-*infected vs *Nosema*-free bees) | |
|  | Mean | SD | Mean | SD | F | p | Mean | SD | Mean | SD | F | p | F | p |
| Al | 5.957 | 0.256 | 5.665 | 0.541 | 1.426 | 0.260 | 3.764 | 0.449 | 3.931 | 0.551 | 70.205 | **0.000** | 0.107 | 0.749 |
| As | 0.250 | 0.064 | 0.222 | 0.070 | 0.525 | 0.485 | 0.147 | 0.054 | 0.134 | 0.024 | 11.467 | **0.005** | 0.996 | 0.336 |
| B | 23.810 | 0.172 | 22.737 | 0.995 | 3.437 | **0.049** | 24.350 | 0.527 | 23.935 | 0.179 | 4.469 | 0.054 | 3.870 | 0.071 |
| Ca | 101.335 | 4.844 | 98.792 | 2.877 | 1.223 | 0.295 | 83.715 | 1.302 | 83.350 | 0.608 | 143.484 | **0.000** | 0.894 | 0.362 |
| Cd | 0.052 | 0.013 | 0.050 | 0.008 | 0.086 | 0.775 | 0.044 | 0.007 | 0.036 | 0.005 | 10.987 | **0.006** | 1.645 | 0.222 |
| Cr | 0.824 | 0.032 | 0.798 | 0.041 | 1.494 | 0.250 | 0.663 | 0.018 | 0.657 | 0.007 | 118.094 | **0.000** | 1.331 | 0.269 |
| Cu | 0.195 | 0.059 | 0.209 | 0.077 | 0.124 | 0.732 | 0.164 | 0.017 | 0.182 | 0.021 | 2.108 | 0.170 | 0.561 | 0.467 |
| Fe | 6.353 | 0.281 | 6.338 | 0.198 | 0.011 | 0.918 | 4.332 | 0.060 | 4.234 | 0.081 | 604.185 | **0.000** | 0.549 | 0.472 |
| Hg | 0.374 | 0.101 | 0.367 | 0.123 | 0.012 | 0.914 | 0.346 | 0.063 | 0.366 | 0.026 | 0.016 | 0.902 | 0.045 | 0.835 |
| K | 496.887 | 8.768 | 502.500 | 8.172 | 1.316 | 0.278 | 182.950 | 8.719 | 182.500 | 7.991 | 7043.354 | **0.000** | 0.334 | 0.573 |
| Mg | 18.273 | 0.685 | 18.653 | 1.079 | 0.531 | 0.483 | 12.075 | 0.254 | 12.350 | 0.067 | 441.708 | **0.000** | 1.046 | 0.325 |
| Mn | 0.872 | 0.011 | 0.882 | 0.015 | 1.871 | 0.201 | 0.229 | 0.006 | 0.224 | 0.008 | 13022.076 | **0.000** | 0.271 | 0.611 |
| Na | 5.832 | 1.567 | 6.084 | 1.406 | 0.086 | 0.775 | 5.003 | 0.540 | 5.666 | 0.275 | 0.927 | 0.353 | 0.613 | 0.448 |
| Ni | 0.593 | 0.019 | 0.584 | 0.033 | 0.355 | 0.564 | 0.462 | 0.009 | 0.466 | 0.003 | 138.937 | **0.000** | 0.001 | 0.980 |
| P | 46.013 | 0.769 | 46.538 | 1.084 | 0.936 | 0.356 | 28.303 | 0.335 | 28.183 | 0.203 | 2822.666 | **0.000** | 0.275 | 0.609 |
| Pb | 0.562 | 0.344 | 0.560 | 0.356 | 0.000 | 0.990 | 0.620 | 0.183 | 0.611 | 0.200 | 0.222 | 0.645 | 0.135 | 0.719 |
| S | 41.180 | 1.446 | 42.303 | 3.926 | 0.432 | 0.526 | 27.815 | 2.086 | 29.613 | 2.486 | 121.673 | **0.000** | 1.746 | 0.209 |
| Se | 0.303 | 0.127 | 0.298 | 0.146 | 0.003 | 0.954 | 0.316 | 0.080 | 0.360 | 0.101 | 0.124 | 0.731 | 0.256 | 0.621 |
| Si | 128.167 | 1.785 | 124.212 | 3.007 | 7.674 | **0.020** | 102.850 | 1.103 | 101.175 | 1.352 | 528.917 | **0.000** | 7.863 | **0.015** |
| Sr | 0.501 | 0.122 | 0.652 | 0.282 | 1.453 | 0.256 | 0.808 | 0.028 | 0.765 | 0.009 | 6.509 | **0.024** | 0.459 | 0.510 |
| V | 0.702 | 0.195 | 0.687 | 0.306 | 0.010 | 0.924 | 0.528 | 0.078 | 0.553 | 0.109 | 3.574 | 0.081 | 0.042 | 0.841 |
| Zn | 6.479 | 2.567 | 6.837 | 2.564 | 0.058 | 0.814 | 4.485 | 0.809 | 4.036 | 1.192 | 8.986 | **0.010** | 0.000 | 1.000 |

**S1c Table. The bioelement content in the winter stores.**

|  | | NI | | NF | | One-way ANOVA results for NI vs NF | |
| --- | --- | --- | --- | --- | --- | --- | --- |
|  | Mean | | SD | Mean | SD | F | p |
| Al | 3.764 | | 0.449 | 3.931 | 0.551 | 0.219 | 0.656 |
| As | 0.147 | | 0.054 | 0.134 | 0.024 | 0.206 | 0.666 |
| B | 24.350 | | 0.527 | 23.935 | 0.179 | 2.223 | 0.187 |
| Ca | 83.715 | | 1.302 | 83.350 | 0.608 | 0.258 | 0.630 |
| Cd | 0.044 | | 0.007 | 0.036 | 0.005 | 3.141 | 0.127 |
| Cr | 0.663 | | 0.018 | 0.657 | 0.007 | 0.340 | 0.581 |
| Cu | 0.164 | | 0.017 | 0.182 | 0.021 | 1.647 | 0.247 |
| Fe | 4.332 | | 0.060 | 4.234 | 0.081 | 3.738 | 0.101 |
| Hg | 0.346 | | 0.063 | 0.366 | 0.026 | 0.369 | 0.566 |
| K | 182.950 | | 8.719 | 182.500 | 7.991 | 0.006 | 0.942 |
| Mg | 12.075 | | 0.254 | 12.350 | 0.067 | 4.395 | 0.081 |
| Mn | 0.229 | | 0.006 | 0.224 | 0.008 | 1.146 | 0.326 |
| Na | 5.003 | | 0.540 | 5.666 | 0.275 | 4.784 | 0.071 |
| Ni | 0.462 | | 0.009 | 0.466 | 0.003 | 0.704 | 0.433 |
| P | 28.303 | | 0.335 | 28.183 | 0.203 | 0.375 | 0.563 |
| Pb | 0.620 | | 0.183 | 0.611 | 0.200 | 0.004 | 0.952 |
| S | 27.815 | | 2.086 | 29.613 | 2.486 | 1.227 | 0.310 |
| Se | 0.316 | | 0.080 | 0.360 | 0.101 | 0.475 | 0.516 |
| Si | 102.850 | | 1.103 | 101.175 | 1.352 | 3.685 | 0.103 |
| Sr | 0.808 | | 0.028 | 0.765 | 0.009 | 8.722 | **0.026** |
| V | 0.528 | | 0.078 | 0.553 | 0.109 | 0.137 | 0.724 |
| Zn | 4.485 | | 0.809 | 4.036 | 1.192 | 0.389 | 0.556 |

**S1d Table. The bioelement content in the apiary soil.**

|  | Mean | SD |
| --- | --- | --- |
| Al | 12290 | 1638 |
| As | 48.04 | 3.513 |
| B | 300.07 | 24.510 |
| Ca | 1 405.6 | 653.2 |
| Cd | 1.634 | 0.268 |
| Cr | 19.51 | 4.054 |
| Cu | 13.79 | 1.627 |
| Fe | 11940 | 2178 |
| Hg | 0.16 | 0.0716 |
| K | 8 681 | 112.8 |
| Mg | 735.8 | 13.52 |
| Mn | 301.2 | 78.98 |
| Na | 4015.67 | 1.281 |
| Ni | 22.16 | 6.325 |
| P | 80.04 | 13.21 |
| Pb | 28.02 | 2.756 |
| S | 2975 | 101.35 |
| Se | 7.75 | 0.694 |
| Si | 72426.67 | 403.7 |
| Sr | 40.13 | 8.156 |
| V | 16.83 | 3.025 |
| Zn | 52.26 | 2.270 |
