# Supplementary material for: Changes in the bioelement content of summer and winter western honeybees (Apis mellifera) induced by Nosema ceranae infection
Source: PLoS One. 2018 Jul 25;13(7):e0200410. doi: 10.1371/journal.pone.0200410 (PMC6060561; doi:10.1371/journal.pone.0200410)
Supplement: S2 Table — Statistica (version 12.0, StatSoft Inc., USA), at the significance level of α = 0.05. Factors: Nosema infection status and bee type. NHS–Nosema health status (Nosema-infected vs. Nosema-free). WBT–worker bee type (summer vs. winter). WBT*NHS–interaction of the Nosema infection status vs worker-bee type. Insignificant effects–printed in bold type. (DOCX) [file pone.0200410.s002.docx]

**S2 Table. Results of the two-way ANOVA, factors: *Nosema* infection status and worker bee type.**

| Bioelement | Factor | Sum of squers | Variance; test F | p; level of significance |
| --- | --- | --- | --- | --- |
| Al | WBT | 329.135 | 11307.598 | 0.0000 |
|  | NHS | 19.233 | 660.762 | 0.0000 |
|  | WBT*NHS | 9.139 | 313.960 | 0.0000 |
| B | WBT | 904.543 | 48.060 | 0.0001 |
|  | NHS | 71509.588 | 3799.407 | 0.0000 |
|  | WBT*NHS | 463.950 | 24.650 | 0.0011 |
| Cu | WBT | 156.508 | 15691.463 | 0.0000 |
|  | NHS | 38.024 | 3812.316 | 0.0000 |
|  | WBT*NHS | 16.145 | 1618.683 | 0.0000 |
| Fe | WBT | 752.083 | 3184.770 | 0.0000 |
|  | NHS | 72.324 | 306.264 | 0.0000 |
|  | WBT*NHS | 195.536 | 828.017 | 0.0000 |
| P | WBT | 9980745.601 | 7938.790 | 0.0000 |
|  | NHS | 364879.688 | 290.229 | 0.0000 |
|  | WBT*NHS | 335838.021 | 267.129 | 0.0000 |
| S | WBT | 17404.083 | 39.198 | 0.0002 |
|  | NHS | 1166256.750 | 2626.704 | 0.0000 |
|  | WBT*NHS | 207770.083 | 467.951 | 0.0000 |
| Si | WBT | 195126.003 | 120.460 | 0.0000 |
|  | NHS | 4424059.203 | 2731.165 | 0.0000 |
|  | WBT*NHS | 733.203 | 0.453 | **0.5200** |
| Sr | WBT | 1864260.130 | 3105.759 | 0.0000 |
|  | NHS | 1840720.535 | 3066.543 | 0.0000 |
|  | WBT*NHS | 1869624.437 | 3114.695 | 0.0000 |
| V | WBT | 0.002 | 3.708 | **0.0903** |
|  | NHS | 0.001 | 2.306 | **0.1674** |
|  | WBT*NHS | 0.001 | 1.659 | **0.2337** |
| Ca | WBT | 7494.206 | 55.813 | 0.0001 |
|  | NHS | 7269.996 | 54.143 | 0.0001 |
|  | WBT*NHS | 2113.006 | 15.737 | 0.0041 |
| K | WBT | 5384120.333 | 946.243 | 0.0000 |
|  | NHS | 52801.333 | 9.280 | 0.0159 |
|  | WBT*NHS | 1425541.333 | 250.535 | 0.0000 |
| Mg | WBT | 104402.708 | 183.648 | 0.0000 |
|  | NHS | 4230.008 | 7.441 | 0.0259 |
|  | WBT*NHS | 17840.941 | 31.383 | 0.0005 |
| Na | WBT | 95926.201 | 1126.533 | 0.0000 |
|  | NHS | 231.441 | 2.718 | **0.1378** |
|  | WBT*NHS | 92348.108 | 1084.513 | 0.0000 |
| Cr | WBT | 0.198 | 44.727 | 0.0002 |
|  | NHS | 0.004 | 0.905 | **0.3692** |
|  | WBT*NHS | 0.055 | 12.300 | 0.0080 |
| Mn | WBT | 130.265 | 18588.417 | 0.0000 |
|  | NHS | 5.423 | 773.854 | 0.0000 |
|  | WBT*NHS | 31.179 | 4449.207 | 0.0000 |
| Se | WBT | 1.342 | 15.791 | 0.0041 |
|  | NHS | 0.173 | 2.039 | **0.1911** |
|  | WBT*NHS | 0.144 | 1.697 | **0.2289** |
| Zn | WBT | 1242.368 | 3491.267 | 0.0000 |
|  | NHS | 819.062 | 2301.705 | 0.0000 |
|  | WBT*NHS | 2.746 | 7.716 | 0.0240 |
| Ni | WBT | 3.075 | 3222.993 | 0.0000 |
|  | NHS | 1.924 | 2017.027 | 0.0000 |
|  | WBT*NHS | 2.250 | 2357.892 | 0.0000 |
| Cd | WBT | 0.033 | 1878.421 | 0.0000 |
|  | NHS | 0.011 | 601.888 | 0.0000 |
|  | WBT*NHS | 0.005 | 266.532 | 0.0000 |
| Hg | WBT | 0.037 | 159136.883 | 0.0000 |
|  | NHS | 0.001 | 2207.521 | 0.0000 |
|  | WBT*NHS | 0.001 | 5443.691 | 0.0000 |
| Pb | WBT | 0.639 | 73.666 | 0.0000 |
|  | NHS | 1.643 | 189.537 | 0.0000 |
|  | WBT*NHS | 0.604 | 69.656 | 0.0000 |
| As | WBT | 0.234 | 924556.661 | 0.0000 |
|  | NHS | 0.003 | 12727.266 | 0.0000 |
|  | WBT*NHS | 0.008 | 31673.056 | 0.0000 |
