# Supplementary figures and images for: Changes in the bioelement content of summer and winter western honeybees (Apis mellifera) induced by Nosema ceranae infection
Source: PLoS One. 2018 Jul 25;13(7):e0200410. doi: 10.1371/journal.pone.0200410 (PMC6060561; doi:10.1371/journal.pone.0200410)

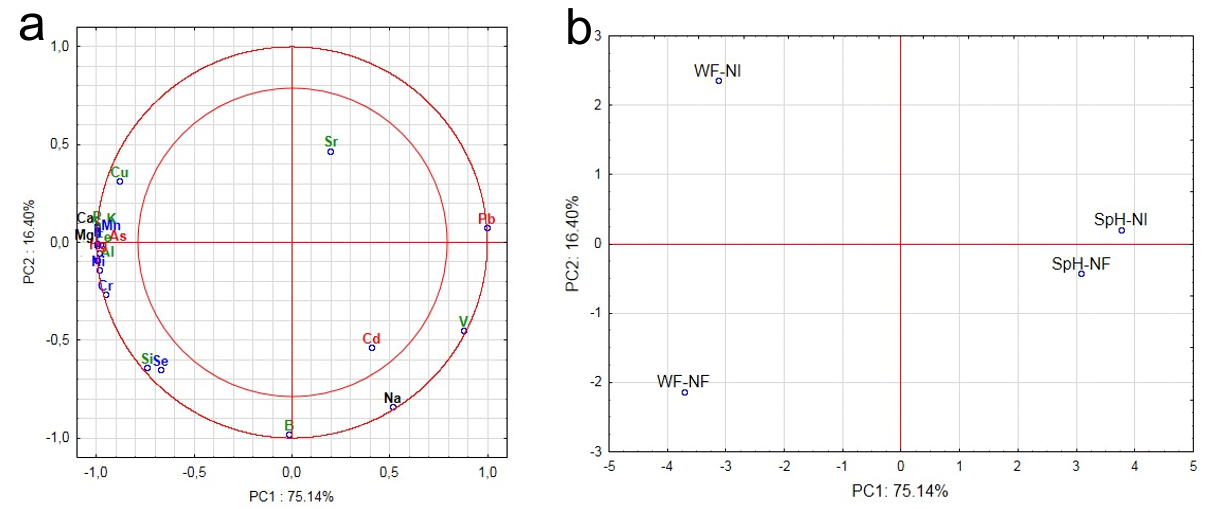

Supplement: S1 Fig — SpH-NI spring honey made by Nosema-infected bees, SpH-NF spring honey made by Nosema-free bees, WF-NF winter food stored by Nosema-infected bees, WF-NI winter food stored by Nosema-free bees. (a) A variable graph showing the position of the load vectors relative to the first two principal components; physiologically essential bioelements are marked in green (Al, B, Cu, Fe, P, S, Si, Sr, and V), electrolytic in black (K, Na, Ca, Cl, and Mg), enzymatic in blue (Cr, Mn, Se, Zn, and Ni) and exclusively toxic in red (Cd, Hg, Pb, and As). (b) The graph shows a strong correlation of bioelement content with seasons (summer, winter), and further with a Nosema infection. (TIF) [file pone.0200410.s003.tif]

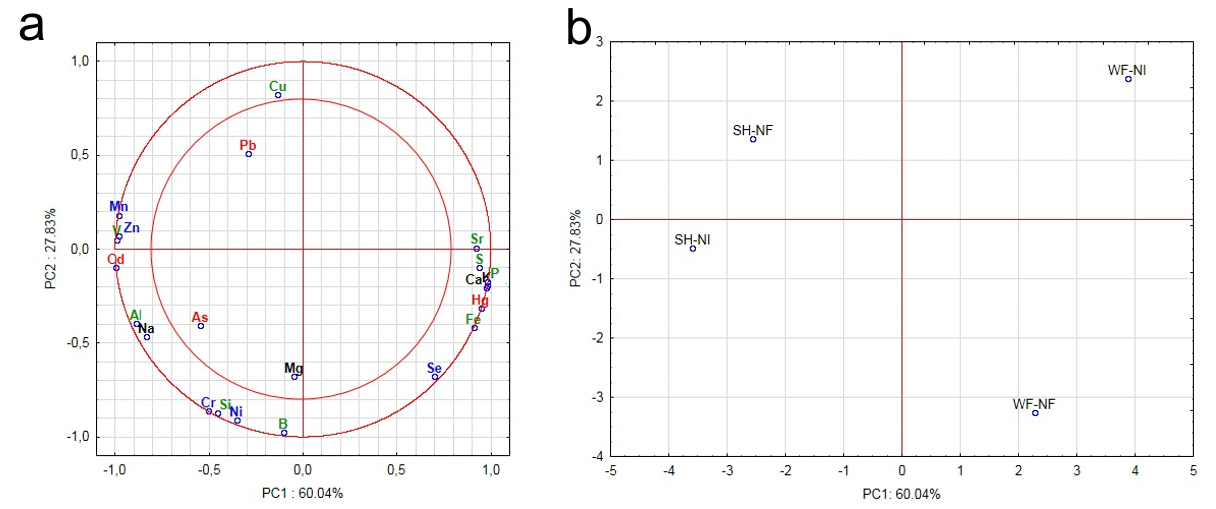

Supplement: S2 Fig — SH-NF summer honey made by Nosema-infected bees, SH-NI summer honey made by Nosema-free bees, WF-NF winter food stored by Nosema-infected bees, WF-NI winter food stored by Nosema-free bees. (a) A variable graph showing the position of the load vectors relative to the first two principal components; physiologically essential bioelements are marked in green (Al, B, Cu, Fe, P, S, Si, Sr, and V), electrolytic in black (K, Na, Ca, Cl, and Mg), enzymatic in blue (Cr, Mn, Se, Zn, and Ni) and exclusively toxic in red (Cd, Hg, Pb, and As). (b) The graph shows a strong correlation of bioelement content with seasons (summer, winter), and further with a Nosema infection. (TIF) [file pone.0200410.s004.tif]
